# Supplementary material for: Shatavari Supplementation in Postmenopausal Women Improves Handgrip Strength and Increases Vastus lateralis Myosin Regulatory Light Chain Phosphorylation but Does Not Alter Markers of Bone Turnover
Source: Nutrients. 2021 Nov 27;13(12):4282. doi: 10.3390/nu13124282 (PMC8708006; doi:10.3390/nu13124282)
Supplement: Supplementary file 1 [file nutrients-13-04282-s001.zip › nutrients-1477395-supplementary.pdf]

Shatavari Supplementation in Postmenopausal Women Improves Handgrip Strength and Increases *Vastus Lateralis* Myosin Regulatory Light Chain Phosphorylation But Does Not Alter Markers of Bone Turnover: A Randomised Controlled Trial – Supplementary Material

Supplementary Methods

Immunoblotting

20 mg skeletal muscle was homogenised in screw top microcentrifuge tubes containing 3 steel beads (3.2mm diameter) and 400µL ice-cold radioimmunoprecipitation assay (RIPA) buffer (50 mM Tris HCl, 150 mM NaCl, 1.0 % (v/v) NP-40, 0.5 % (w/v) Sodium Deoxycholate, 1.0 mM EDTA, 0.1 % (w/v) sodium dodecyl sulfate [SDS], 0.01 % (w/v) sodium azide, protease inhibitor cocktail, phosphatase inhibitor cocktail, pH of 7.4). Homogenisation was performed using a Speedmill Plus homogeniser (Analytik Jena AG, Jena, Germany). Lysates were then removed to a different microcentrifuge tube and centrifuged at 14,000 g for 10 min at 4 °C. The supernatant was removed to another microcentrifuge tube. The total protein concentration of each cell lysate supernatant was quantified by a bicinchoninic acid assay (Thermo Scientific), according to the manufacturer's instructions. Lysates were stored at - 80 °C prior to use in downstream applications.

Total protein lysates of known concentration were mixed 3:1 with 4x Laemmli sample loading buffer (0.5M Tris-HCl pH 6.8, 20 % (v/v) Glycerol, 10 % (w/v) SDS, 0.1 % (w/v) bromophenol blue, 10 % (v/v) beta-mercaptoethanol [βME]) and water to generate polyacrylamide gel loading stocks of known concentration. Samples were boiled at 100°C for 5 min. Pre-cast polyacrylamide gels (Mini-PROTEAN® TGX™ Precast Gels, Bio-Rad) were loaded with an appropriate volume of loading stock (see Supplementary Table 1 for details of loading mass, gel percentages etc). Samples from both supplementation conditions were run side by side on each gel.

Proteins were transferred onto polyvinylidene difluoride (PVDF) membrane (Amersham Hybond-P, GE Healthcare, Buckinghamshire, UK) in an electroblotting buffer (0.025M Tris base, 0.192M glycine, 20 % (v/v) methanol at pH 8.4) using the 'Standard SD' program of the Trans-Blot Turbo semi-dry transfer system (Bio-Rad, CA, USA). Membranes were blocked for 1 h and agitated overnight with primary antibody diluted in TBS-T at 4 °C (Table). The membranes were washed for 4 x 5 min in 1xTBS-T (50 mM Tris hydrochloride [Tris-HCl], 150 mM sodium chloride [NaCl], 0.1% (v/v) Tween 20 at pH 7.5) and agitated for 1 h at room temperature with horseradish peroxidase-conjugated secondary antibody (Table). TBS-T washes were repeated and the membranes incubated with ECL Prime Western blotting detection reagents (Amersham Biosciences, Amersham, UK) according to the manufacturer's instructions. Bands were visualised on the ChemiDoc MP imaging system (Bio-Rad). Blots were washed briefly in TBS-T and stained with 0.1% (w/v) coomassie blue solution (Methanol (50% [v/v], glacial acetic acid 10% [v/v] H_2O 40% [v/v]) to ensure even protein loading. An open-source, public domain software package (Image J v1.47) was used for band quantification.

Supplementary Table S1: Immunoblotting Antibody Details

Target Protein	SDS Page Gel %	Protein Load (µg)	Blocking Solution (5% w/v)	Primary Antibody Details (catalogue number, supplier)	Primary Antibody Dilution in TBS-T	Secondary Antibody Details/ Dilution in TBS-T
**Akt total**	12%	20	Milk	Akt (9272, CST)	1:3000	
**Akt Ser^{473}**	12%	20	BSA	Phospho-Akt Ser473 (4060T, CST)	1:3000	
**p70S6k**	12%	20	Milk	p70S6k (9202, CST)	1:5000	
**p70S6k^{Thr389}**	12%	20	BSA	Phospho-p70 S6 Kinase Thr389 (9234, CST)	1:5000	
**4EBP1**	4-20%	30	Milk	4EBP1 (9452, CST)	1:2500	
**4EBP1$^{Thr37/46}$**	4-20%	30	Milk	Phospho-4E-BP1 Thr37/46 (9459, CST)	1:2500	
**S6**	4-20%	30	Milk	S6 Ribosomal Protein (5G10, CST)	1:2500	
**S6Ser$^{240/244}$**	4-20%	30	Milk	Phospho-S6 Ribosomal Protein Ser 240/244 (5364, CST)	1:2500	Goat Anti-Rabbit IgG H&L (HRP) (ab205718) IgG antibody (Abcam, Cambridge, UK)
**MLC**	12%	30	Milk	Anti-Myosin Light Chain 2 antibody (ab79935, Abcam)	1:2500	1: 15000
**MLC^{ser20}**	12%	30	Milk	Anti-Myosin light chain (phospho S20) antibody (ab2480, Abcam)	1:2500	
**Col1a1**	4-20%	20	Milk	Anti-COL1A1 antibody [N1N2], N-term (GTX112731, Genetex)	1:2500	
**RUNX2**	4-20%	20	Milk	RUNX2 (#12556, CST)	1:2500	

Sodium dodecyl-sulphate polyacrylamide gel electrophoresis (SDS-PAGE), Tris-buffered saline with 0.1% Tween ® 20 Detergent (TBS-T), Bovine Serum Albumin (BSA), Horseradish peroxidase (HRP), Cell Signalling Technology (CST)

Enzyme Linked Immunosorbent Assays

Supplementary Table S2: ELISA Details

Target Protein	Product Name	Catalogue Number	Manufacturer
**M-CSF**	Human M-CSF DuoSet ELISA	DY216	R&D Systems
**OPG**	Human Osteoprotegerin/TNFRSF11B DuoSet ELISA	DY805	R&D Systems
**IL-6**	IL-6 Human Uncoated ELISA Kit	88-7066-22	20
**IL-1β**	IL-1 beta Human Uncoated ELISA Kit	88-7261-22	20

Macrophage colony-stimulating factor (M-CSF), osteoprotegerin (OPG), interleukin 6 (IL-6), interleukin 1 beta (IL-1β)

Supplementary Results

Participant baseline outcomes

Participant baseline strength characteristics are outlined in Supplementary Table 3. There were no significant baseline strength differences between the supplementation groups.

Supplementary Table S3: Participant Baseline Characteristics

Outcome	Placebo	Shatavari	P value
**Age**	66.8 ± 1.5	70.1 ± 2.1	0.23(t-test)
**BMI**	24 ± 1.5	22.5 ± 0.9	0.41(t-test)
**Handgrip Strength (kg, mean ± SD)**	23.1 ± 4.2	21.7 ± 5.0	0.5 (t-test)
**Maximum Voluntary Contraction (N mean ± SD)**	104.9 ± 31.1	109.0 ± 16.4	0.72 (t-test)
**Maximum Isokinetic Concentric Force (N median ± IQR)**	81.1 ± 46.4	82.0 ± 21.9	0.85 (Mann Whitney U)
**Maximum isokinetic**	111.1 ± 27.1	106.7 ± 31.6	0.97 (Mann

**Eccentric Force (N median ± IQR)**		Whitney U)

Myosin Regulatory Light Chain Phosphorylation

The spread of myosin regulatory light chain phosphorylation data with the lowest datapoint in the placebo dataset removed is presented in Supplementary Figure 1. These data are included to allow the reader to appreciate the spread of other data. No valid reason for exclusion of the lowest datapoint from analyses was identified.

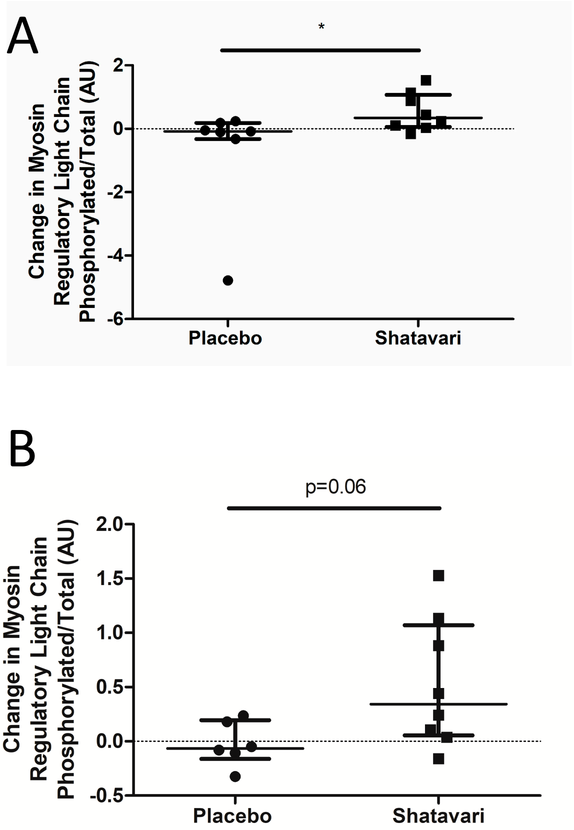

Supplementary Figure S1. Shatavari Supplementation Increases Vastus Lateralis Myosin Regulatory Light Chain Phosphorylation in Postmenopausal Women. Postmenopausal women supplemented with placebo (N = 10) or shatavari (N = 10) for 6 weeks. Total myosin regulatory light chain (MLC) and myosin regulatory light chain phosphorylation (pMLC) were quantified via immunoblotting at baseline and at 6 weeks. Samples were assayed in duplicate. Data are presented as difference scores with the median ± interquartile range of these differences. A) Full dataset B) spread of data with the lowest datapoint in the placebo dataset removed.

Full Versions of Immunoblots presented in the Manuscript

A

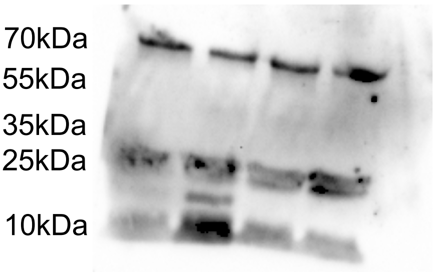

Full pMLC Blot presented in main paper (cut, prior to antibody probing, other half presented in B)

B

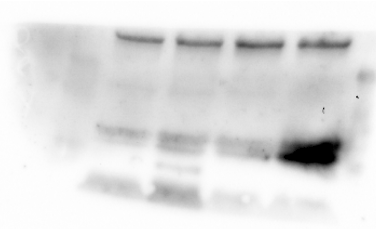

Same samples as A developed under different conditions, illustrating reproducibility of pMLC relative results

C

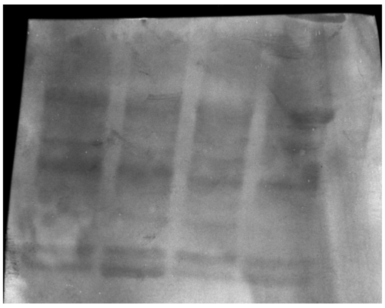

Full pMLC Coomassie blot

D

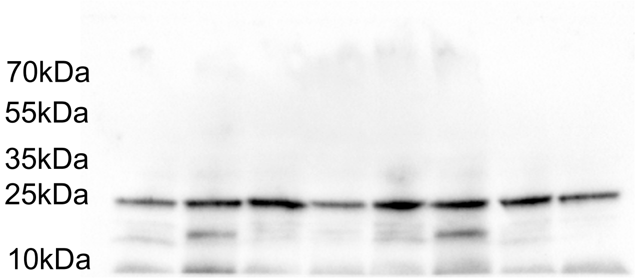

Full MLC Blot presented in main paper (right 4 lanes presented)

E

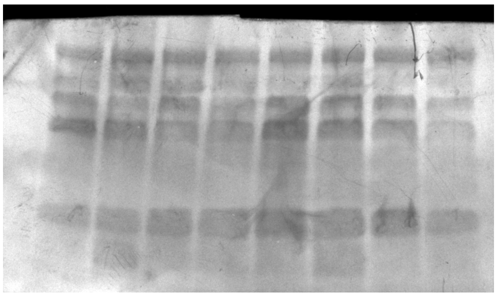

Full MLC Coomassie blot

Supplementary Figure S2. Full Versions of Myosin Light Chain Immunoblots Presented in the Manuscript

Full blots are provided principally to allow the reader to observe that quantified bands were produced at the correct molecular weight, free from proximal abberent bands. However for context, samples were run in the order: placebo pre – placebo post – shatavari pre – shatavari post x 2
Some blots display an additional positive control sample, its position is indicated by an * Where band signals on one side of the blot were not optimal (e.g. B), suggesting poor antibody or substrate coverage, the duplicate assay was redone for quantification purposes.

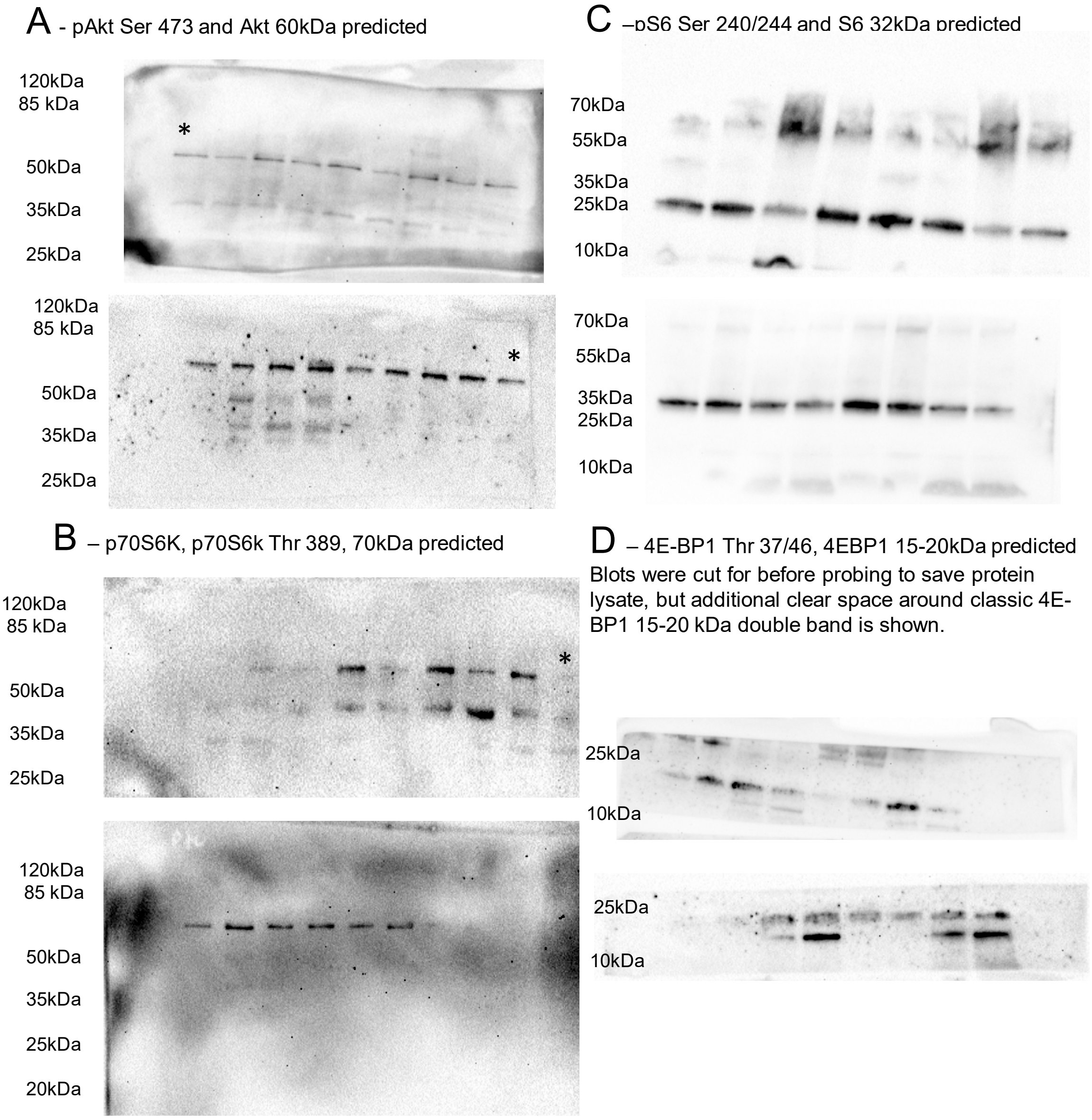

Supplementary Figure S3. Full Versions of Other Immunoblots Presented in the Manuscript
